# Lipoatrophy/lipohypertrophy outcomes after antiretroviral therapy switch in children in the UK/Ireland

**DOI:** 10.1371/journal.pone.0194132

**Published:** 2018-04-04

**Authors:** Steve Innes, Justin Harvey, Intira Jeannie Collins, Mark Fredric Cotton, Ali Judd

**Affiliations:** 1 Family Infectious Diseases Clinical Research Unit (FAMCRU), Stellenbosch University, Cape Town, South Africa; 2 Department of Paediatrics and Child Health, Tygerberg Children’s Hospital and Stellenbosch University, Cape Town, South Africa; 3 Centre for Statistical Consultation, Stellenbosch University, Cape Town, South Africa; 4 Medical Research Council Clinical Trials Unit at University College, London, United Kingdom; Columbia University Medical Center, UNITED STATES

## Abstract

**Background:**

Following widespread use of stavudine, a thymidine analogue, in antiretroviral therapy (ART) over the past three decades, up to a third of children developed lipoatrophy (LA) and/or lipohypertrophy (LH). Following phasing-out of stavudine, incidence of newly-diagnosed LA and LH declined dramatically. However, the natural history of existing cases should be explored, particularly with prolonged protease inhibitor exposure.

**Methods:**

The Collaborative HIV Paediatric Study (CHIPS) is a multicentre cohort study of most HIV-infected children in the United Kingdom and Ireland. Those on ART with a LA/LH assessment recorded in 2003–2011 were included. Assessments were completed annually by consultant physicians. Using the 0–3 grading system, LA or LH was defined as grade 2 or 3. Resolution was defined as return to grade 1 or 0 in all body regions.

**Results:**

Of 1345 children followed for median (IQR) 5.5 (2.9, 8.2) years after ART initiation, 30 developed LA and 27 developed LH, all at least 2 years after ART initiation. Median age at LA diagnosis was 11 (10, 13) years and at LH diagnosis was 13 (11, 15) years. Children with LA were more likely white (p<0.0001); lower height-for-age z-score at ART initiation (p = 0.02); initiated ART earlier (p = 0.04), with longer ART exposure (p = 0.04). Children with LH were similar to those without. Analysis of individual drugs revealed that LA was associated with greater duration of exposure to stavudine and didanosine; while LH was associated with greater duration of exposure to stavudine and ritonavir (given alone or in combination with another protease inhibitor). Median time in follow-up following ART switch was 2.8 (1.9, 4.9) and 2.5 (1.6, 4.7) years respectively. Resolution occurred in 10 (30%) of LA cases (median time to resolution 2.3 [1.8, 3.6] years) and 3 (11%) of LH cases (median time to resolution 2.0 [1.7, 2.1] years).

**Conclusions:**

Prevalence of LA and LH were low, with some resolution noted, especially for LA. More long-term data are needed.

## Introduction

Antiretroviral therapy (ART)-related lipoatrophy (LA) is a disfiguring loss of subcutaneous fat, particularly in the face, limbs and buttocks [1]. ART-related lipohypertrophy (LH) is abnormal accumulation of metabolically-active intra-abdominal visceral fat, breast fat and fat in the nape of the neck [1]. LA, when recognizable in the community, may lead to stigmatization [2,3] and poor self-esteem. Adolescents are especially vulnerable and ART non-adherence may be a consequence [4–6].

In the early days of the ART era, LA occurred in up to one-third of children on thymidine analogue ART, mainly stavudine, while LH appears related to protease inhibitors (PI) and efavirenz [7–9]. Subsequently, the World Health Organization recommended phasing out stavudine from first-line ART [10]. While the incidence of *newly-diagnosed* LA has declined dramatically, the natural history of existing cases remains uncertain [11–14]; the effect of prolonged PI exposure on LH is also uncertain.

The mechanism of LA is thought to be due to mitochondrial damage, causing adipocyte apoptosis and adipose tissue loss [15–17]. Thus, extensive adipocyte loss may limit recovery potential and explains why severe LA is usually permanent [7,11]. The mechanism of LH is less well-established but is no longer believed to be due to redistribution of fat lost elsewhere. ART-induced LA and LH were initially assumed to be components of a single “Lipodystrophy Syndrome” [18]. However, mounting data show that they are independent conditions with separate mechanisms, but can occur simultaneously as the same ARVs are implicated [8,19–21]. It is therefore appropriate to report these conditions individually. Historically, paediatric researchers have typically neither analyzed nor presented these two conditions separately, therefore limiting understanding of these conditions. The aim of the present study is to determine the natural history of LA and LH separately in a cohort of children.

## Methods

The Collaborative HIV Paediatric Study (CHIPS) is a multicentre cohort of HIV-infected children in the United Kingdom and Ireland [22]. Its main objectives are to describe clinical, laboratory and treatment outcomes for these children. These data are collected annually in a case report form completed by the attending physician or nurse. Data are captured and stored centrally at the Medical Research Council Clinical Trials Unit at University College London. As of March 2012, the CHIPS database collected data on 1,791 children since its inception in 2000. Of these, 1,188 remained in active follow-up. CHIPS is a national cohort of all children diagnosed with HIV in the UK and Ireland, it has National Health Service Research Ethics approval allowing for a waiver of individual consent to ensure complete coverage.

Children were eligible for inclusion in this analysis if on ART and had at least one LA or LH assessment between 2003 and 2011. The assessments were performed by a consultant physician using a standardized visual grading scale, allowing analysis of changed assessment over time. The CHIPS data collection form provided four options (none, mild, moderate and severe) for grading of LA or LH. These correlate with the standard grade 0, 1, 2, 3 system (18, 19, 23) as follows: None (grade 0); Mild—Noticeable only if specifically looked for with no change in clothes fitting other than for normal growth (equivalent to grade 1); Moderate—Easily noted by patient or clinician with clothing becoming tight or loose not due to normal growth (equivalent to grade 2); Severe—Obvious to the casual observer or required a change in clothing due to change in body shape not due to normal growth (equivalent to grade 3). The norm in existing literature, and followed in the present analysis, is to combine grade 2 or 3 as unequivocal cases [18,23,24]. Fat loss was graded separately for the face, arms and legs, along with prominence of veins. Diagnosis of LA required grade 2 or 3 changes in any of those four body regions. Similarly, fat accumulation due to LH was graded separately at each of three body regions: posterior neck; breasts; and abdomen, requiring grade 2 or 3 changes in any of those regions. Recovery of LA and LH was defined as return to grade 1 or 0 in all body regions. LA and LH were assessed separately. Children had only one LA/LH assessment recorded per calendar year.

Chi-square test compared categorical variables and two-sample T-test or Wilcoxon Rank Sum test compared continuous variables in cases of LA or LH vs patients without LA/ LH as appropriate. Kaplan Meier was used to estimate time to LA or LH after start of ART, among all those eligible for inclusion in the analysis and censored at time of first LA/LH diagnosis, death or last visit. Logistic regression modelling was used to assess association between specific antiretroviral drug exposures and lipoatrophy or lipohypertrophy, adjusting for age, gender and ethnicity. Individual ARV drugs associated with the outcomes in univariate analysis (p<0.10) were included in the multivariate analysis following a backward stepwise approach, variables with p-values <0.05 were included in the final model. This was performed for lipoatrophy and lipohypertrophy separately. Infrequently used drugs (n<100) were not included due to their small sample size. Among patients with a LA or LH event, time to recovery was estimated, and patients were at risk from date of LA or LH diagnosis and censored at the earliest of the following: date of resolution, death or last visit in paediatric care. Fisher’s Exact test compared categorical variables among cases. Children with only one LA or LH assessment available did not contribute to the time to recovery analysis.

## Results

Of 1,345 patients, median follow-up time after ART start was 5 (interquartile range, IQR 3, 8) years. Overall prevalence of LA at any time was 2.2% (30/1,345), and LH 2.0% (27/1,345). Nine patients (6 male) had both LA and LH diagnosed, of whom 4 had both conditions recognized simultaneously; and LA preceded LH for 5 patients. The cumulative median duration of ART exposure at first LA/LH diagnosis was over 6 years (Table 1). The most commonly used initial regimen was 2NRTI+NNRTI based regimens (in 62% of children included in the analysis). The most common initial treatment combination was 3TC+ABC+EFV used in 228 children (17% of 1335 with initial ART regimen data), followed by ZDV+3TC+NVP (n = 137, 10%) and ZDV+3TC+ABC+NVP (n = 101, 7.6%). Among patients with LA or LH, the most common initial regimen was ZDV+ddI (dual therapy) (in 19%), and ZDV+3TC+ABC (in 10%). Notably, only 16% of patients were diagnosed with LA/LH while on their initial regimen while 84%were diagnosed while on subsequent regimens.

**Table 1:** Description of cases of lipoatrophy (LA) and lipohypertrophy (LH) compared to non-cases.

		LA ever diagnosed n = 30	LH ever diagnosed n = 27	LA/LH never diagnosed n = 1303	*P* (LA vs. no LA/LH)	*P* (LH vs. no LA/LH)
		n (%) or median (IQR)				
**Gender**	**male**	17 (57%)	18 (67%)	634 (49%)	0.46	0.08
**Ethnicity**	**Black African**	13 (43%)	19 (70%)	1015 (78%)	**<0.0001**	0.41
	**White**	7 (24%)	4 (15%)	109 (8%)		
	**Other ethnicities**	10 (33%)	4 (15%)	179 (14%)		
**Age at ART initiation (years)**		3.9 (1.3, 6.5)	6 (3.4, 10.6)	5.8 (1.7, 10.3)	**0.04**	0.41
**Age at LA/LH diagnosis (years)**		11 (10, 13)	13 (11, 15)	-	-	-
**Cumulative ART exposure prior to LA or LH diagnosis (years)**		7.9	6.2	6.0**	**0.04**	0.45
		(5.7, 12.0)	(2.8, 11.4)	(2.9, 9.8)		
**Weight-for-age Z-score at ART initiation**		-1	-0.1	-0.4	0.16	0.33
		(-1.7, +0.4)	(-1.4, +0.6)	(-1.3, +0.4)		
**Height-for-age Z-score at ART initiation**		-1.5	-0.8	-0.8	**0.02**	0.76
		(-2.3, -0.8)	(-2.0, +0.3)	(-1.6, +0.0)		
**Body mass index at ART initiation**		16.8	17.9	16.8	0.64	0.09
		(15.5, 18.1)	(15.8, 19.6)	(15.5, 18.5)		

** Cumulative duration of exposure prior to last recorded visit in those with no LA or LH

**Fig 1:**
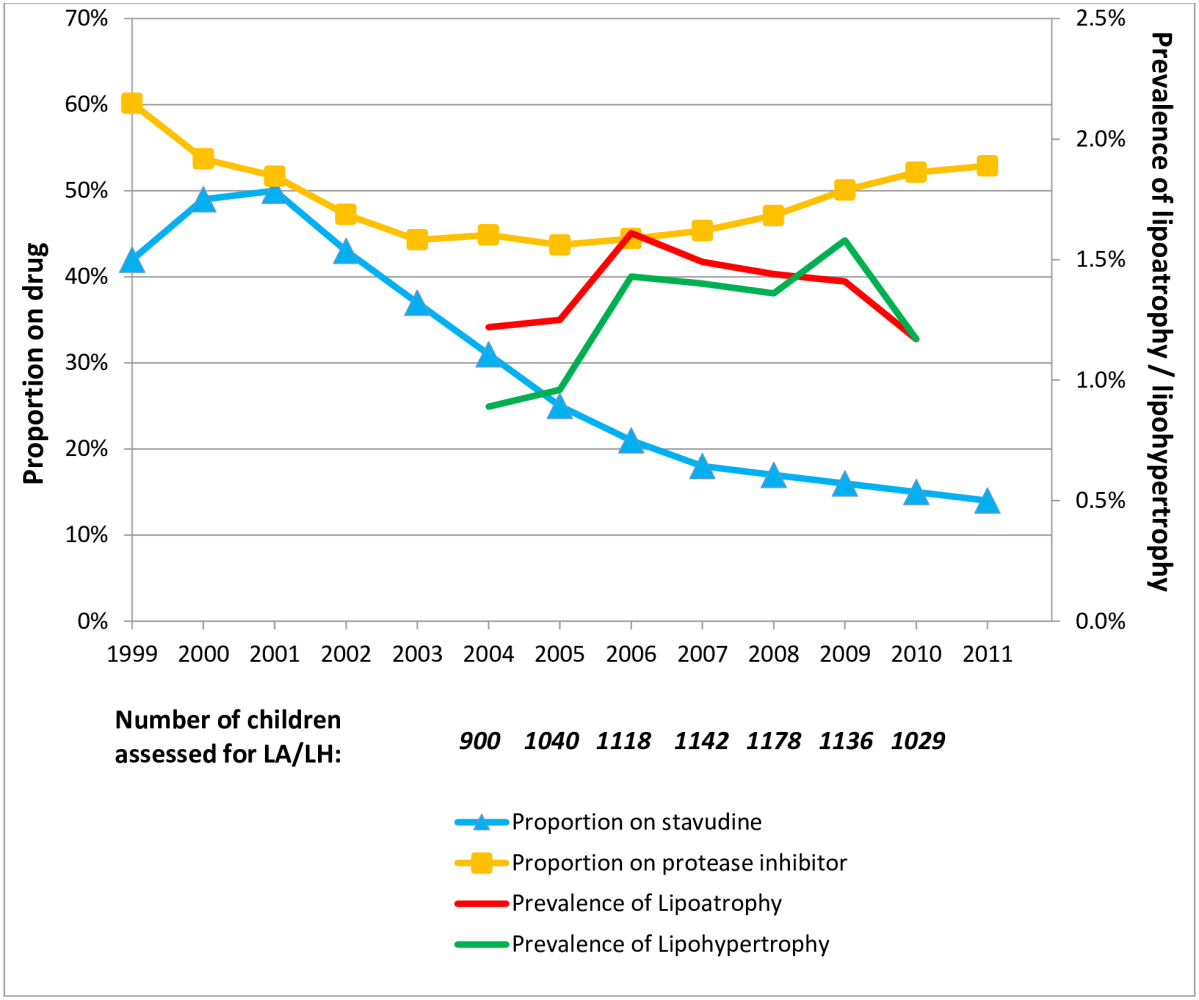
Prevalence of lipoatrophy (LA) and lipohypertrophy (LH) and proportion of children on stavudine and on protease inhibitors during each calendar year in the UK and Ireland. The denominator for calculation of prevalence in each calendar year is presented below the graph.

Prevalence of LA and LH remained unchanged despite marked reduction in stavudine use from around 50% in 2000 to 15% in 2010 (Fig 1).

On univariate analysis, children with LA (compared to children without LA) were more likely white, initiated ART earlier, exposed to ART for longer, and lower height-for-age at ART initiation (Table 1), whilst children with LH had similar characteristics to those without LA/LH. Analysis of individual drugs (Tables 2 and 3) revealed that LA was associated with greater duration of exposure to stavudine and didanosine; while LH was associated with greater duration of exposure to stavudine and ritonavir (given alone or in combination with another PI).

**Table 2:** Multivariable model of risk factors associated with Lipoatrophy.

		Odds ratio	95% CI		P>ChiSq
Age at ART initiation (for each additional year)		0.97	0.87	1.08	0.589
Gender (female versus male)		0.73	0.33	1.64	0.449
Ethnicity	Black African	1			
	White	3.24	1.15	9.14	0.026
	Other ethnicities	4.36	1.70	11.15	0.002
Duration of exposure to d4T (for each additional year)		1.36	1.17	1.57	<0.0001
Duration of exposure to ddI		1.16	1.01	1.34	0.033
Duration of exposure to 3TC		0.85	0.73	0.99	0.032

d4T = stavudine; ddI = didanosine; 3TC = lamivudine.

Note: 3TC here is probably a surrogate for total duration of ART exposure, since it was included in the majority of regimens.

**Table 3:** Multivariable model of risk factors associated with Lipohypertrophy.

		Odds ratio	95% CI		P>ChiSq
Age at ART initiation (for each additional year)		1.16	1.05	1.29	0.005
Gender (female versus male)		0.46	0.20	1.08	0.076
Ethnicity	Black African	1			
	White	1.17	0.35	3.90	0.076
	Other ethnicities	1.30	0.42	4.04	0.655
Duration of exposure to d4T (for each additional year)		1.46	1.26	1.70	<0.0001
Duration of exposure to RTVh		1.65	1.16	2.36	0.006
Duration of exposure to RTVl		1.18	1.00	1.40	0.049

d4T = stavudine; RTVI = ritonavir given in combination with another protease inhibitor; RTVh = ritonavir given alone.

**Fig 2:**
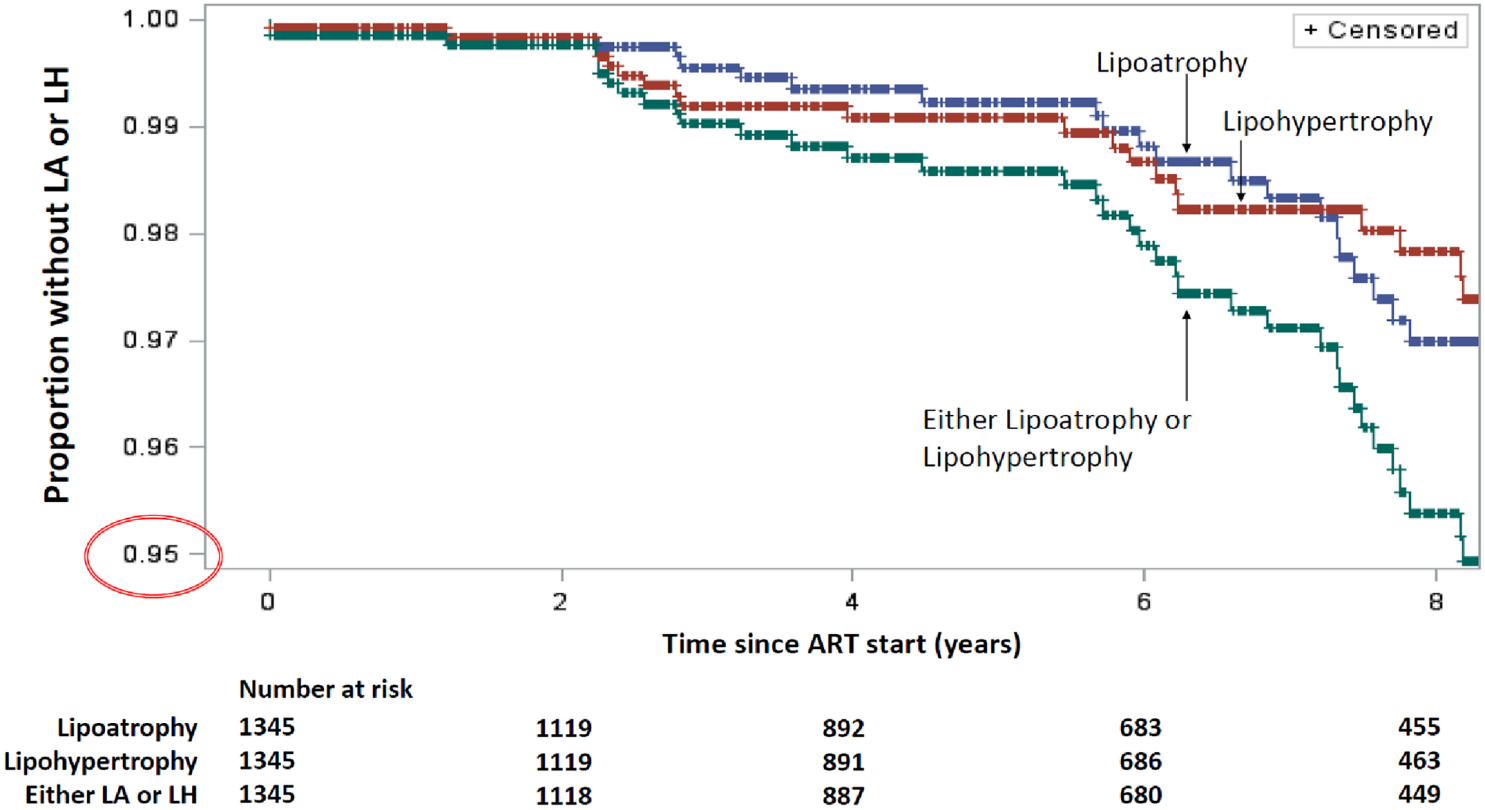
Kaplan-Meier of emergence of LA or LH in children on ART in the UK and Ireland.

While LA was most commonly noticed in the face (74% of cases), limbs were also commonly affected (63%). LH affected the breasts and abdomen with equal frequency (59%) while the nape of the neck was infrequently affected (23%). Recognition of LA and LH occurred from 2 years on ART (Fig 2), with the median duration on ART prior to LA or LH diagnosis being 7.9 (5.7, 12.0) years and 6.2 (2.8, 11.4) years respectively (Table 1).

### Recovery from LA or LH

Median follow-up after LA diagnosis was 2.8 (IQR 1.9, 4.9) years, and after LH diagnosis 2.5 (1.6, 4.7) years. Over this period 10 LA cases (33%) and 3 LH cases (11%) had recovered, with median time to resolution of 2.3 (IQR 1.8, 3.6) and 2.0 (1.7, 2.1) years respectively.

## Discussion

Despite the large proportion of children in the UK and Ireland ever exposed to stavudine and with prolonged use of PIs, the prevalence of LA and LH was low and constant over time, with children diagnosed after a median of 6 years of cumulative ART exposure. Few cases resolved: only one-third with LA and fewer of those with LH. Similar to previous studies of recovery of ART-related LA and LH, our sample size was small but represents one of the first longitudinal studies with standardized data collection in a multi-centre setting with national coverage.

Despite inconsistencies of definition, the prevalence of LA and LH (when defined by visual grading) appear to be consistently higher in reports from lower income compared to high income settings [7,25,26]. Stavudine use in the UK/Ireland declined substantially after 2000, but continued to be used in almost all children in less developed settings due to its availability, low cost and early incorporation into fixed drug combination tablets [27,28].

The CHIPS cohort benefits from a long duration of follow-up, with the cases reported having a median of over 6 years cumulative ART exposure at time of LA/LH diagnosis. Most paediatric HIV cohorts in resource-limited settings have <5 years of follow-up. Factors associated with LA in our study include white ethnicity, also reported in other European paediatric cohorts [8], and prolonged exposure to ART. Although previous studies have highlighted the increased risk of LA with prolonged exposure to stavudine and didanosine [23,29], we wanted to investigate the potential contribution of other drugs including PIs which are increasingly used as paediatric cohorts mature. There is a need to continue monitoring for LA/LH which may yet emerge with prolonged ART exposure.

### Recovery of lipoatrophy and lipohypertrophy

Very little paediatric data are available on recovery of LA or LH [11–14], probably because recovery is slow. Rates of recovery depend heavily on the threshold used for diagnosis, with the least severe changes having the highest chance of recovery [7,12–14,30]. Grade 2 is the most clinically appropriate threshold for LA because the diagnosis is not in doubt, yet the changes are not yet noticeable to the general public. Studies that included grade 1 changes in their case definition (which by definition implies some uncertainty or subtlety), report higher levels of recovery. Aurpibul et al (2012) incorporated minor grade 1 changes in their definition of LA and reported resolution in 16 (73%) of 22 paediatric patients with LA and 14 (47%) of 30 patients with LH [13]. In contrast Aurpibul’s findings, Vigano et al (2007) found that, while the level of new fat accrual measured by DXA had normalized in 24 paediatric patients two years after switching from stavudine, pre-existing visible LA changes remained static [14]. Using a visual grading scale, Sawawiboon et al (2012) found improvement in LA in 11 (23%) of 48 affected children 4 years after switching from stavudine, although complete resolution occurred in only one child [12]. Similarly, we found a low level of resolution for both LA (33%) and LH (11%) after a median of 2½ years of follow-up.

### Limitations

In CHIPS the diagnosis of LA and LH were based on subjective assessment by many physicians. Significant variation in diagnostic practice across clinics may have occurred. In addition, reporting bias due to increasing physician awareness over time may have occurred. ART regimens usually contain a minimum of three effective drugs and, although certain drugs are commonly coupled, the number of possible combinations is large. To tease out the independent effect of each drug is difficult as the potential for confounding by association is high. The current analysis could not adequately assess the influence of multiple risk factors on LA or LH resolution due to the small number of cases. Despite puberty being a well-known risk factor, Tanner stage was often not recorded and so could not be evaluated. Including age as a variable may not have adequately compensated for this deficiency.

## Conclusion

Prevalence of LA and LH were low, with some resolution noted, especially for LA. More long term data are needed.
